# Characteristics of lipid micro- and nanoparticles based on supercritical formation for potential pharmaceutical application

**DOI:** 10.1186/1556-276X-8-386

**Published:** 2013-09-13

**Authors:** Islane Espírito Santo, André São Pedro, Rosana Fialho, Elaine Cabral-Albuquerque

**Affiliations:** 1 PEI (Programa de Engenharia Industrial) - Escola Politécnica, Universidade Federal da Bahia, Rua Prof. Aristides Novis, 02, Federação, Salvador, Bahia 40210-630, Brazil

**Keywords:** Liposomes, Solid lipid nanoparticles, Supercritical carbon dioxide

## Abstract

The interest of the pharmaceutical industry in lipid drug delivery systems due to their prolonged release profile, biocompatibility, reduction of side effects, and so on is already known. However, conventional methods of preparation of these structures for their use and production in the pharmaceutical industry are difficult since these methods are usually multi-step and involve high amount of organic solvent. Furthermore, some processes need extreme conditions, which can lead to an increase of heterogeneity of particle size and degradation of the drug. An alternative for drug delivery system production is the utilization of supercritical fluid technique. Lipid particles produced by supercritical fluid have shown different physicochemical properties in comparison to lipid particles produced by classical methods. Such particles have shown more physical stability and narrower size distribution. So, in this paper, a critical overview of supercritical fluid-based processes for the production of lipid micro- and nanoparticles is given and the most important characteristics of each process are highlighted.

## Review

### Introduction

Nowadays, the utilization of supercritical fluid-based technology is considered as a promising substitute to the traditional methods of particle production since it is an efficient and environment-friendly technique. Supercritical fluids are defined as substances for which both temperature and pressure are above critical values. Beyond this point, the liquid and gas phases become indistinguishable because the densities of the phases are identical, and only a homogeneous medium exists [1].

Supercritical fluids have many industrial applications, including chemical reactions, extraction of essential oils, supercritical chromatography, manufacturing of semiconductors, micronization of pharmaceutical excipients, production of drug delivery systems, and so on [2,3]. The most widely used supercritical fluid in drug delivery applications is carbon dioxide (CO_2_) because of a low critical temperature of 304 K and a moderate critical pressure of 7.3 MPa. It is nonflammable, nontoxic, and environment friendly; it is miscible with a variety of organic solvents and is readily recovered after processing. It is also a small and linear molecule and thus diffuses faster than conventional liquid solvents.

Supercritical carbon dioxide (scCO_2_) offers a wide range of possible applications in the pharmaceutical field [4], which allows the processing of bioactive compounds under mild operation conditions avoiding their degradation [5]. The use of CO_2_ as solvent or raw material has been investigated in academia and/or industry since 1950 and has intensified 30 years later with the implementation of large-scale plants using online systems [6]. The approaches for processing bioactive compounds include mainly the particle size reduction of bulk products to nanometer scale [7] and association of drug molecules to particulate carriers [8].

CO_2_ molecule possesses no dipole moment, which means that it is nonpolar and, when it is in supercritical state, CO_2_ can be a good solvent to solubilize nonpolar substances. However, CO_2_ possesses a quadrupole moment, which enables the dissolution of some polar and slightly polar compounds at high pressures [9,10]. So, the scCO_2_ presents a substantial solubility on polymers and lipids, typical drug carriers. The solubilization of scCO_2_ promotes decrease in viscosity of the molten drug carrier, making possible their bombing through the plant [11].

Other significant advantages of supercritical fluid processing include its nonflammability, its relative low cost, the possibility of its total recycling, the production of organic solvent-free particles, the achievement of particulate systems with a narrow particle size distribution, and the its one-step operation. Furthermore, all processes run into a closed system facilitating the establishment of an ascetical production of sterile formulations [6,12,13].

### Liposomes

Liposomes are colloidal associations of amphiphilic lipids that organize themselves spontaneously in bilayer vesicles as a result of unfavorable interactions between phospholipids and water. As they have lipophilic and hydrophilic portions, liposomes can entrap substances with varying lipophilicities in the phospholipid bilayer, in the aqueous compartment, or at the bilayer interface [14-16] which can modify physicochemical properties and enhance the biological activity of the compounds [17].

As liposomes are composed of phospholipids, they have interesting physical and chemical properties, such as osmotic activity, permeability of their membranes to different solutes, and also the capacity of interacting with membranes of different cell types [18]. They also have the ability of minimizing side effects of drugs, protecting them from degradation, specific targeting, and biocompatibility [19].

Selecting the method of liposome production is related to the materials or the lipid composition of the vesicles that will be used. The starting point for all conventional methods of liposome production is the dissolution of phospholipids in an organic solvent, and the main difference between these methods is the way in which the lipid membrane is dispersed in aqueous media [20-25]. These methods have some drawbacks in common, such as the large number of steps needed to produce the vesicles, the utilization of a large amount of organic solvent in the beginning or during the process, the lack of diameter size uniformity and, moreover, the low stability of produced particles [26]. To overcome these drawbacks, the utilization of supercritical fluid is an alternative to produce these nanoparticles.

### Liposome production by scCO_2_ processing

As aforementioned, supercritical fluid technology is an interesting alternative for the production of safer and more stable drug delivery particles. Indeed, the utilization of supercritical fluid technology in the production of liposomes entrapping pharmaceuticals and biopharmaceuticals is a promising field under intense investigation. Table 1 summarizes different methods to produce liposomes using supercritical fluids.

**Table 1:** Different supercritical fluid methods utilized for liposomes production

**Method**	**Phospholipid composition**	**Active ingredient**	**Particle size**	**Reference**
Supercritical liposome method	Phosphatidylcholine, phosphatidylserine, and cholesterol	FITC-dextran and TSZnPc	~200 nm	[27]
Rapid expansion of supercritical solution process	Phosphatidylcholine and cholesterol	*Atractylodes macrocephala* essential oil	~173 nm	[28]
Depressurization of an expanded solution into aqueous media	Diastearoylphosphatidylcholine and cholesterol	-	50 to 200 nm	[29]
Solution-enhanced dispersion by supercritical fluid process	Soy phospholipids	Puerarin	1 μm	[30,31]
Gas anti-solvent process	Soy phospholipids	-	-	[31]
	Phosphatidylcholine and cholesterol	Amphotericin B	0.5 to 3 μm	[32]
Aerosol solvent extraction system	Phosphatidylcholine and cholesterol	Miconazole	DNS	[33]
Supercritical anti-solvent process	Lecithins S20, S75, and S100	-	1 to 40 μm	[34]
	Lecithin S75	-	1 to 40 μm	[35]
	Lecithin S75	Fluorescent markers	0.1 to 100 μm	[26]
	Hydrogenated soy phosphatidylcholine, soy phosphatidylcholine, and cholesterol	Docetaxel	200 to 300 nm	[36]
	Hydrogenated soy phosphatidylcholine	Vitamin D_3_	1 μm	[37]
	Hydrogenated soy phosphatidylcholine	Lutein	200 to 500 nm	[38]
Continuous anti-solvent process	Soy lecithin	-	0.1 to 100 μm	[39,40]
Supercritical reverse-phase evaporation	Dipalmitoylphosphatidylcholine	Glucose and cholesterol	0.1 to 1.2 μm	[41]
	Phosphatidylcholine, phosphatidylethanolamine, phosphatidylinositol, and phosphatidic acid	Glucose and cholesterol	0.1 to 1.2 μm	[42]
	Phosphatidylcholine and dioleoylphosphatidylcholine	Glucose	0.1 to 1.2 μm	[14]
Improved supercritical reverse-phase evaporation	Dipalmitoylphosphatidylcholine	Glucose	1.5 μm	[43,44]

DNS, data not shown.

#### ***Supercritical liposome method***

**Figure 1:**
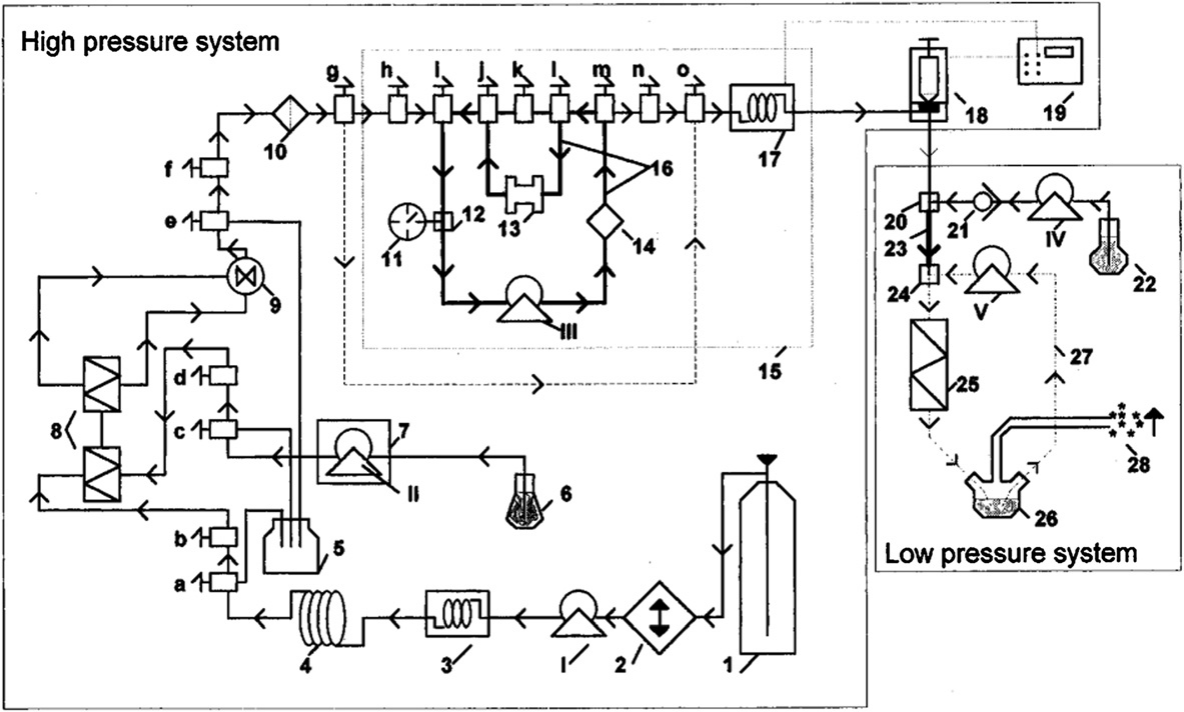
**Schematic representation of the apparatus utilized by Frederiksen et al. **[27]. Composed of a (I) CO_2_ pump, (II) modifier pump, (III) high-pressure recycling pump, (IV, 4) pulse dampener capillary, (V) low-pressure recycling pump, (1) CO_2_ cylinder, (2) cooling device, (3, 11) manometer, (5) waste flask, (6) measuring cylinder, (7) pump T-piece, (9) dynamic mixer, (10) filter, (12, 20, 24) T-piece, (13) cartridge guard column, (14) UV detector, (15) Plexiglas water bath, (16) high-pressure recycling system, (17) pressuring transducer, (18) back-pressure regulator, (19) pressure controller, (21) checking valve, (23) encapsulation capillary, (25) static mixer, (26) liposomal suspension reservoir, (27) low-pressure recycling system, and (28) fume cupboard to remove CO_2_; a, b, c, d, e, f, g, h, i, k, j, l, m, n, and o are valves.

Frederiksen et al. [27] created a laboratory method aiming to produce liposomes encapsulating water-soluble compounds utilizing scCO_2_ as an alternative to utilizing large amounts of organic solvents. The apparatus developed for this method is depicted in Figure 1 and it is divided into two parts: a high-pressure and a low-pressure system that possess a recycling loop each and are connected by a capillary. The presence of this capillary before the low-pressure system allows the addition of the aqueous solution in the bulk of phospholipid solution, which increases the encapsulation of water-soluble compounds into liposomes. Briefly, phospholipids and cholesterol were added into the high-pressure system and dissolved in scCO_2_ and ethanol. Then they were kept in the recycling loop for 30 min at 25 MPa and 333 K to ensure an effective dissolution of the lipids and guarantee a homogeneous solution. After that, the solution was led to the low-pressure system in order for it to expand. According to the authors, there is formation of foam during the expansion of the supercritical fluid in the presence of the aqueous solution. In order to suppress the foam formation, a static mixer was added to the recycling loop. Thereafter the expansion, lipids were precipitated, brought in contact with the aqueous solution, and kept in the recycling loop for other 30 min in order to form liposomes. Liposomes obtained by this process presented a bimodal distribution with an average size of 200 nm, and this method used 15 times less organic solvent to get the same encapsulation efficiency as conventional techniques. However, the encapsulation efficiency of hydrophilic compounds in liposomes was about 15%, which is about 50% less than the encapsulation of water-soluble compounds in liposomes made by DRV or reverse-phase evaporation methods. Due to the complexity of this process, there are no other studies involving liposome production by this method.

#### ***Rapid expansion of supercritical solution process***

Rapid expansion of supercritical solution (RESS) process consists of the saturation of scCO_2_ with the solute followed by a rapid expansion of the solution through a heated nozzle to a low-pressure chamber. The rapid expansion/decompression is achieved by allowing passage through a nozzle at supersonic speeds. The decrease of the pressure forces the evaporation of CO_2_, leading to the supersaturation and then precipitation of the solid that is collected from the gaseous stream [45,46].

**Figure 2:**
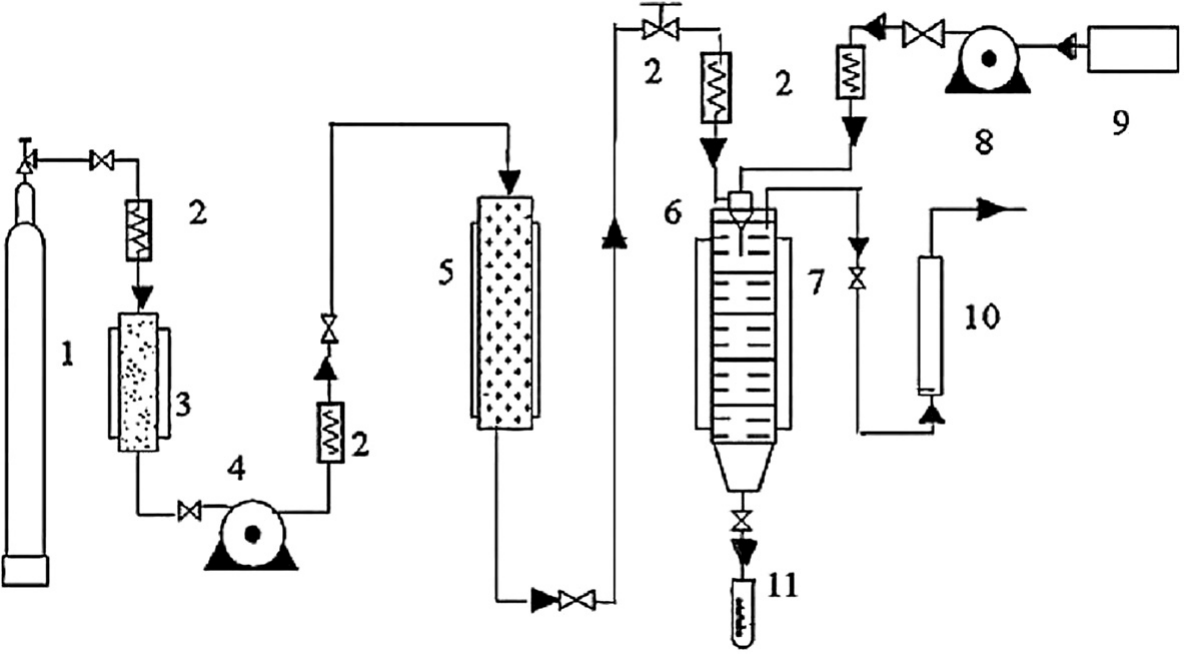
**Schematic representation of the RESS apparatus used by Wen et al**. [28]**to produce liposomes.** In this apparatus, the following are found: (1) CO_2_ cylinder, (2) heat exchanger, (3) refrigerating machine, (4, 8) syringe pump, (5) reactor, (6) coaxial injector, (7) collector, (9) storage tank, (10) rotameter, and (11) volumetric cylinder.

This supercritical process is not suitable to produce these lipid vesicles because (1) phospholipids are not completely soluble in pure scCO_2_ and (2) liposomes can only be completely formed in an aqueous medium. Thus, Wen et al. [28] developed some modifications in the conventional RESS process to produce liposomes. The schematic representation of the apparatus is depicted in Figure 2. Phosphatidylcholine, cholesterol, and the essential oil of *Atractylodes macrocephala* Koidz were dissolved in a mixture of scCO_2_/ethanol, and after the system reached equilibrium, a buffer solution was injected by a syringe pump into the dissolved solutes. The final mixture was expanded through a nozzle into the collector to evaporate CO_2_. According to the authors, liposomes formed by this method presented good physicochemical characteristics and a higher encapsulation efficiency was obtained with pressures up to 20 MPa, temperatures of 323 to 338 K, and ethanol mole fractions of 5% to 15% in scCO_2_. The optimization of the method provided liposomes with spherical morphology, narrow size distribution with an average size of 173 nm, and encapsulation efficiency of 82.18% at 30 MPa, 338 K, and ethanol amount of 15%.

#### ***Depressurization of an expanded solution into aqueous media***

**Figure 3:**
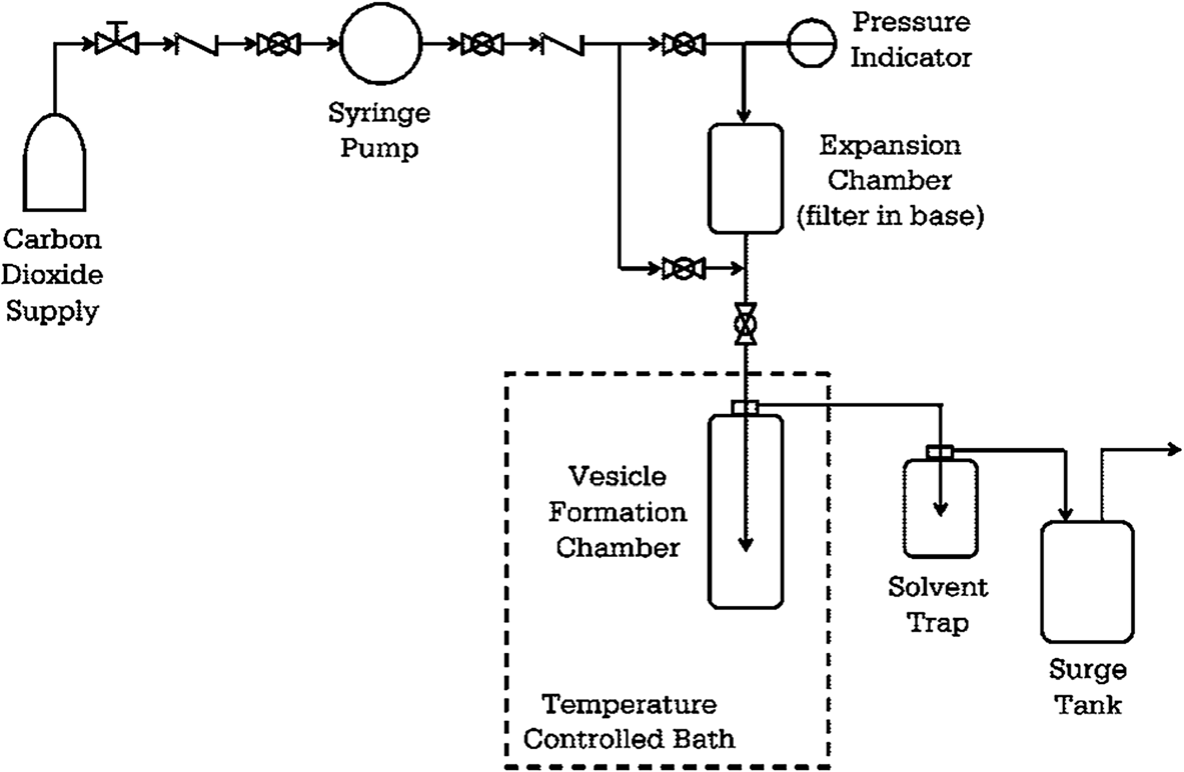
**Apparatus utilized for DESAM process developed by Meure et al. **[29].

Meure et al. [29] developed a process (depressurization of an expanded solution into aqueous media (DESAM)) that can remove almost every organic solvent added into the system and also works at mild conditions - moderate temperatures and pressures below 6 MPa. In this technique, a fast and simple process for bulk liposome formation was developed. Phospholipids were initially dissolved in organic solvents - ethanol or chloroform. Then, CO_2_ was sparged into the system with a syringe pump in order to form an expanded lipid solution inside the expansion chamber. This expansion occurs because the gas rapidly diffuses into the solution, promoting the phenomenon. After that, the expanded lipid solution was atomized through a nozzle into a heated aqueous media. When ethanol was utilized to dissolve the lipids, the expansion chamber parameters were 295 K and 5.0 to 5.5 MPa, while the parameters were 294 K and 3.8 to 4.0 MPa when chloroform was utilized. According to the authors, the residual solvent concentration was less than 4% *v*/*v* in all liposome preparations. This value is less than another supercritical method that had values of residual solvent volume fraction of 14% to 17% *v*/*v*[27].The apparatus depicted in Figure 3 was utilized to perform the experiments of liposome production from 50 to 200 nm.

#### ***Solution-enhanced dispersion by supercritical fluid process***

Li et al. [30,31] implemented a method of production of phospholipid complex encapsulating puerarin utilizing solution-enhanced dispersion by supercritical fluid (SEDS) process in a semi-continuous operation. In SEDS process, the supercritical fluid acts not only as an anti-solvent but also as a dispersion medium. The solution is provided from the outer passage and dispersed by the supercritical fluid which is quickly introduced in the inner passage. Due to the presence of a premixing chamber in the inner nozzle, the solution and anti-solvent can be molecularly dispersed before the formation of the solution jet. This contact of supercritical fluid and liquid solution streams leads to the generation of a finely dispersed mixture followed by particle precipitation [2,3]. Furthermore, as it is an efficient single-step, totally enclosed, and easy-to-scale up process, it can produce more homogeneous particles for drug delivery systems.

**Figure 4:**
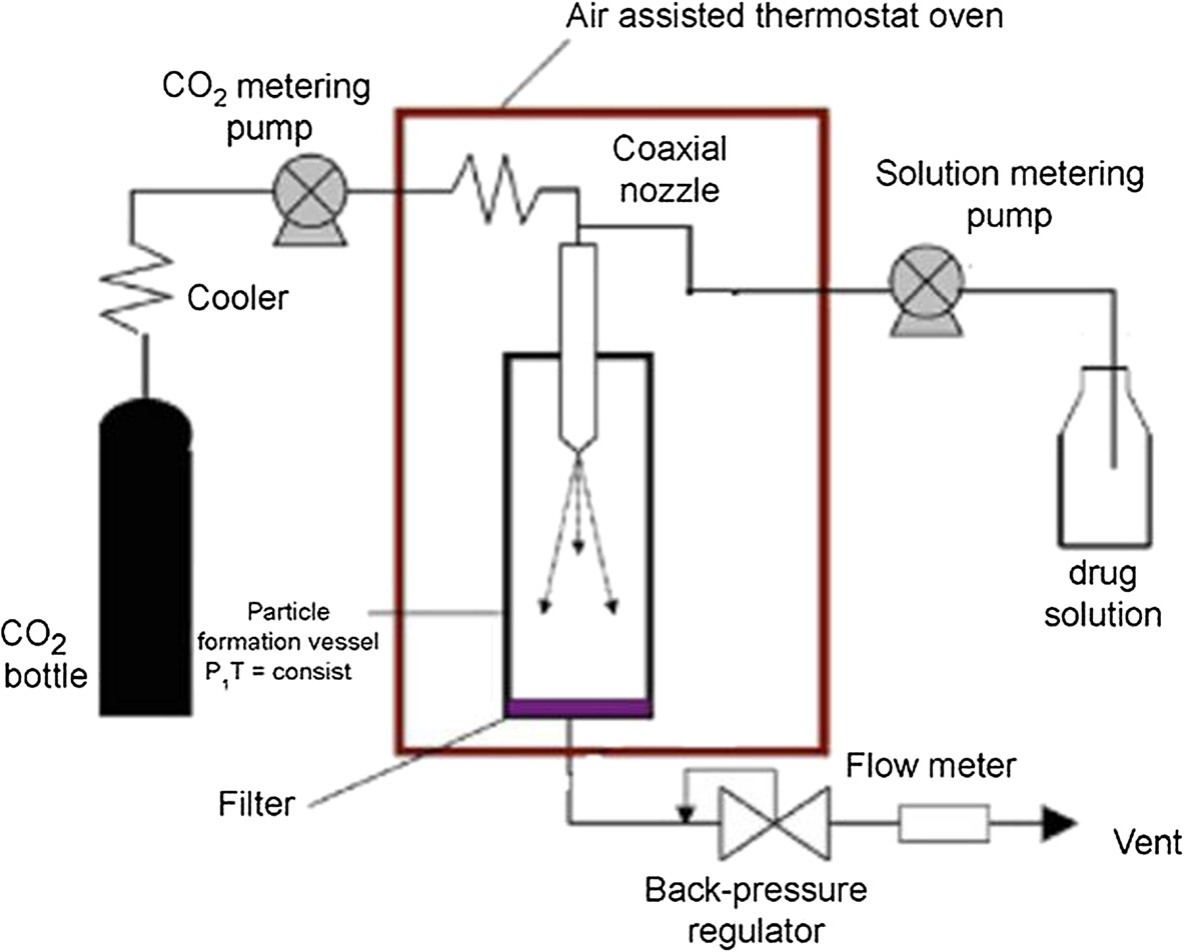
**Representation of the SEDS process apparatus utilized by Li et al**. [31].

So, for liposome production [30,31], phospholipid complex is defined as the presence of active substances inside phospholipid vesicles at solid state. The representation of the apparatus is depicted in Figure 4. Puerarin is an isoflavone and one of the major constituents of *Pueraria lobata* (Willd.) Ohwi, a plant utilized in traditional medicine [47]. Organic liquid solution of puerarin and soy phospholipids was added cocurrently with CO_2_ by two syringe pumps into the particle formation vessel. CO_2_ and the liquid solution were sprayed into the vessel through a coaxial nozzle. A high flow rate of CO_2_ was utilized to promote mixture of the organic solution with scCO_2_. Therein the organic solvents utilized are dispersed from the bulk of the solution, leading to the extraction of the solvents and the precipitation of the particles. A temperature range of 303 to 313 K, pressure range of 8 to 12 MPa, CO_2_ flow rate of 25 to 65 mL min^-1^, and proportion of the solution flow rate to scCO2 from 1% to 5% were chosen by the authors to be the operation parameters, which were optimized at 308 K, 10 MPa, CO_2_flow rate of 45 mL min^-1^, and the solution-to-scCO_2_ flow rate proportion of 1%. Under this optimized conditions, puerarin-phospholipid vesicle complex of 1 μm and agglomerates of 5.93 μm were obtained. This process was shown to be efficient in the production of micrometric phospholipid complexes in just one step. However, the authors did not measure the residual solvent concentration in the particle to ensure that the particles were almost free of solvents.

#### ***Gas anti-solvent process***

In the gas anti-solvent (GAS) process, compressed gas is gradually introduced into a liquid solution. This ability to solubilize large amount of gases is the basis of this technique. This solubilization leads to a volumetric expansion of the liquid phase followed by a decrease of the liquid solvent strength, resulting in the precipitation of small particles of the solute. The major advantage of GAS process is the possibility of processing a wide range of compounds and also the possibility of controlling particle size and distribution. However, as particles are produced in a liquid medium, it requires another stage for drying the particles [48,49].

Taking GAS process characteristics into account, Li et al. [31] also tried to produce a phospholipid complex with puerarin by this method. But instead of using a semi-continuous configuration as used in SEDS process, the plant was utilized in a batch configuration. The apparatus utilized by the authors was the same as depicted in Figure 4 with one modification - the ethanolic or chloroformic liquid solution was added into the particle formation vessel before it was closed, instead of pumping the solution into the chamber. So, one syringe pump was not used to perform this process. After the addition of the solution, the scCO_2_ was pumped into the vessel and left for 3 h without agitation at 10 MPa and 311 K. The flow rate of CO_2_ was maintained constant during the experiment in order to remove the organic solvents of the solution, and the slow depressurization of the system occurred at the same temperature of the experiments. However, this process was not able to produce phospholipid complexes.

In another study, Kadimi et al. [32] produced liposomes at 15.0 MPa and 333 K encapsulating amphotericin B based on the GAS process. The vesicle efficacy was tested against *Aspergillus fumigatus*. Briefly, solutions of phospholipids, chloroform, and methanol were loaded into an autoclave. Then, CO_2_ was pumped till the pressure arrived 15.0 MPa and the temperature was set at 333 K. The compressed CO_2_ was released into the autoclave. After the equilibration period, a saline solution was pumped into the autoclave to induce the liposome formation, and then, the vessel was slowly depressurized. Also, in order to compare the results with different methods, liposomes were also produced by thin-film hydration [21]. Liposomes produced by supercritical technique were smaller (0.15 to 3 μm for GAS method against 0.15 to 6 μm by thin-film hydration), with better morphology and size distribution than the vesicles made by the conventional method. Also, vesicles made by the GAS process presented better antifungal activity against the *A. fumigatus* strain, with an encapsulation efficiency of 25% to 30% of amphotericin B.

#### ***Aerosol solvent extraction system***

Kunastitchai et al*.*[33] applied aerosol solvent extraction system (ASES) process to produce liposomes entrapping miconazole, an imidazole antifungal agent. The production of these liposomes was done in two steps: (1) obtention of a miconazole-phospholipid complex by ASES and (2) further hydration with aqueous phosphate buffer in order to form the phospholipid vesicles. Different amounts of miconazol (19% and 38%) and ratios of phosphatidylcholine/cholesterol (8:2 and 10:0, *w*/*w*) were dissolved in a mixture of methanol/methylene chloride (2:8 *w*/*w*) with or without the addition of poloxamer 407. These solutions were sprayed through a nozzle with a diameter of 0.4 mm into a high-pressure vessel filled with scCO_2_ to remove the organic solvents and precipitate the dried liposomes. In order to optimize the process of liposome formation, temperature, pressure, and CO_2_ density ranges used were 308 to 328 K, 8.5 to 10.5 MPa, and 0.30 to 0.50 g mL^-1^, respectively. The CO_2_ flow rate was 6 kg h^-1^ and spraying rate was 6 mL min^-1^. After the atomization, the solution was washed with scCO_2_ in order to extract the remaining organic solvents. Then, it was hydrated with phosphate buffer at different pH levels (4.0 and 7.2) and submitted to gentle agitation at 328 K. According to the authors, the percentage yield of liposome formation was higher when the temperature used was 308 K and the CO_2_ density was 0.30 g mL^-1^. Therefore, the optimized parameters utilized were 308 K, 8.0 MPa, and 0.30 g mL^-1^.

#### ***Supercritical anti-solvent process***

Supercritical anti-solvent process (SAS) is the most popular precipitation process involving supercritical anti-solvent due to the wide range of compounds that can be used, the control of particle size and distribution, and the facility of adaptation for a continuous operation [3,50]. Basically, the compound is dissolved in a liquid solvent and sprayed to a chamber that already has supercritical fluid, leading to their rapid contact. This contact causes supersaturation of the solution, then fast nucleation, and consequently, diffusion of the anti-solvent in the liquid phase and formation of small particles [51,52].

**Figure 5:**
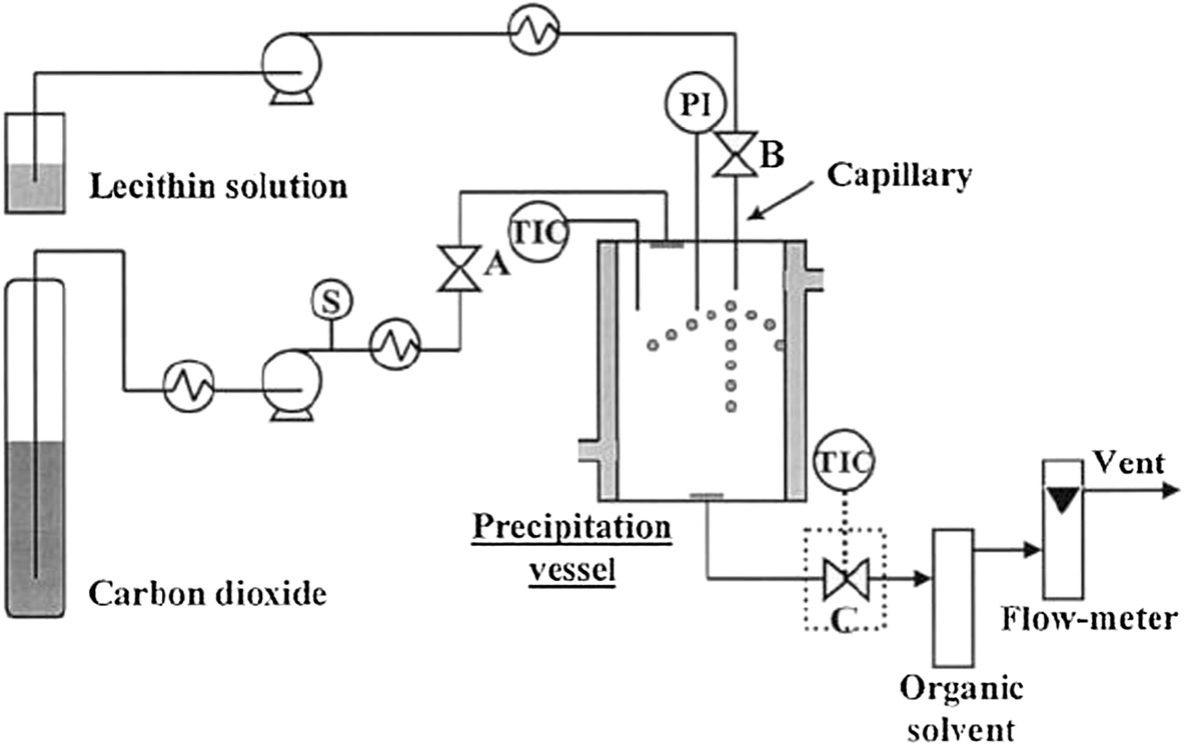
**The SAS apparatus utilized for the production of liposomes**[35].

Badens et al. [34] and Magnan et al. [35] produced liposomes from three different lecithins: S20, S75, and S100. These lecithins contained different amounts of phosphatidylcholine, phosphatidylethanolamine, and phosphatidylinositol. Different operation parameters were analyzed by this study, such as pressure (8.0 to 12.0 MPa), temperature (303 to 323 K), and liquid solution flow rate (10 to 40 mL h^-1^). CO_2_ flow rate value was maintained constant during all the experiments. The vesicle formed had a diameter size between 1 and 40 μm, had a spherical shape, was partly agglomerated, and seemed to be free of solvent, according to infrared analysis. The apparatus that was used for these studies is depicted in Figure 5.

Lesoin et al. [26] compared liposomes produced by SAS and the thin-film hydration methods in an apparatus similar to the one depicted in Figure 5. According to the authors, the vesicles produced by supercritical fluids presented a spherical shape, bimodal size distribution in the range of 0.1 to 100 μm, and encapsulation efficiency of fluorescent markers of 20%. However, the ellipsoidal vesicles made by the traditional method seemed to be more dispersed, but this method has serious issues of reproducibility and repeatability, which makes the supercritical process more attractive than the conventional one.

Another interesting study described the production of PEGylated liposomes using the SAS process to encapsulate docetaxel, one of the most important chemotherapeutic agents against cancer. Hydrogenated soy phosphatidylcholine (PC), soy PC, and cholesterol in different proportions were utilized to produce the vesicles with DSPE-PEG_2000_. The utilization of saturated and unsaturated phospholipids enhanced the liposomal stability in about 3 months with high entrapment efficiency. So, docetaxel and the phospholipids were dissolved in chloroform and methanol. This solution was sprayed into a high-pressure vessel where the operational temperature and pressure were then set. Once the system reached the steady state, the lipid solution was pumped into the chamber that had the scCO_2_ to permit the mixing of the phases and, consequently, precipitating the lipid particles in the vessel. The vesicles formed were small and unilamellar with a size range between 200 and 300 nm. *In vitro* release studies showed that the vesicles presented controlled drug release during 48 h. No residual organic solvent at the end of the preparation was found. The authors concluded that PEGylated liposomes produced by supercritical fluid technology are more stable, have smaller size, and are free from residual organic solvent [36].

Xia et al. [37,38] produced proliposomes using the supercritical anti-solvent process. It was shown that the proliposomes, which are dry free-flowing particles, have a media size of 200 nm with a narrow size distribution. The increased pressure utilized in the system (8.0 to 12.0 MPa) favors the formation of small molecules. After the hydration, the formed liposomes encapsulating lutein had a size of about 500 nm, while vesicles encapsulating vitamin D_3_ presented 1 μm, approximately. The authors affirm that the proliposomes are easily hydrated, producing unilamellar liposomes. The vesicles formed by supercritical fluids have entrapping efficiency of lutein and vitamin D_3_ that reaches 90% each.

#### ***Continuous anti-solvent process***

**Figure 6:**
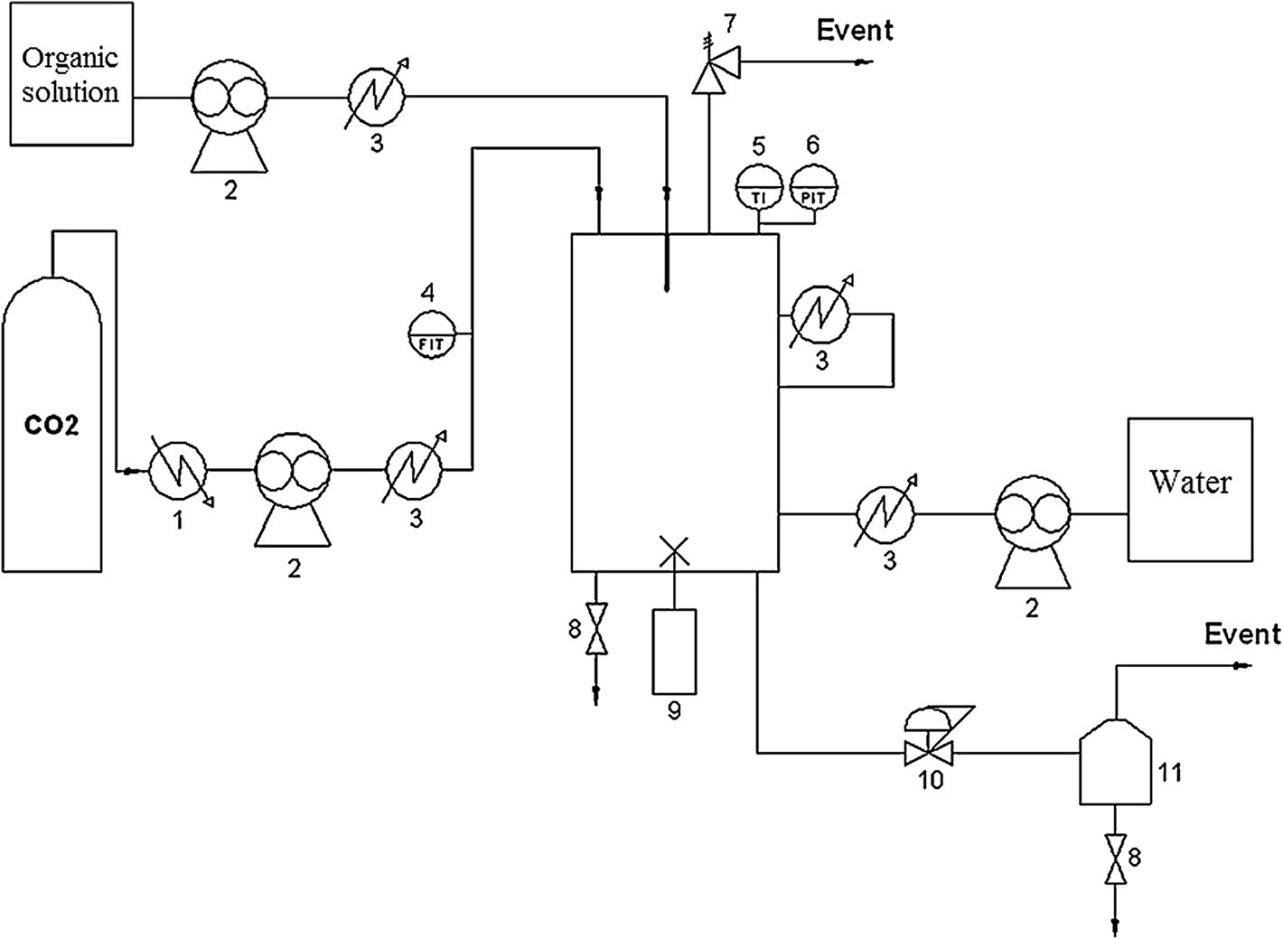
**Schematic representation of the CAS apparatus utilized by Lesoin et al.**[40]. In this apparatus, the following are found: (1) cooler, (2) volumetric pump, (3) heater, (4) flow indicator transmitter, (5) temperature indicator, (6) back-pressure valve, (7) safety valve, (8) release valves, (9) stirring, (10) control valve, and (11) dryer.

Lesoin et al. [39,40] developed a new single-step supercritical process to produce liposomes called continuous anti-solvent process (CAS) (Figure 6). Two different procedures were developed for this method: CAS1 and CAS2. The difference between the processes is the number of exits: while CAS1 is a single-exit process, CAS2 has two exits. In CAS1, an initial amount of aqueous phase was added inside the autoclave followed by the injection of CO_2_. The organic solution was sprayed to the autoclave while the liquid phase was under stirring. When the phases were in equilibrium, a valve at the bottom of the autoclave was opened, releasing the CO_2_ and the liposome suspension. In order to maintain the same amount of liquid inside the autoclave, an aqueous solution was injected in a continuous way. On the other hand, in the CAS2 method, the aqueous phase was added into the autoclave and then it was filled with CO_2_. When the work pressure was reached, the organic solution was added similarly to the CAS1 method. However, when the system seemed to be homogeneous, a valve on the top of the autoclave was opened, releasing the CO_2_, and the liposomal suspension was recovered from the bottom of the vessel. The mean diameter of liposomes produced by the CAS methods ranged from 0.1 to 100 μm.

#### ***Supercritical reverse-phase evaporation and improved supercritical reverse-phase evaporation***

**Figure 7:**
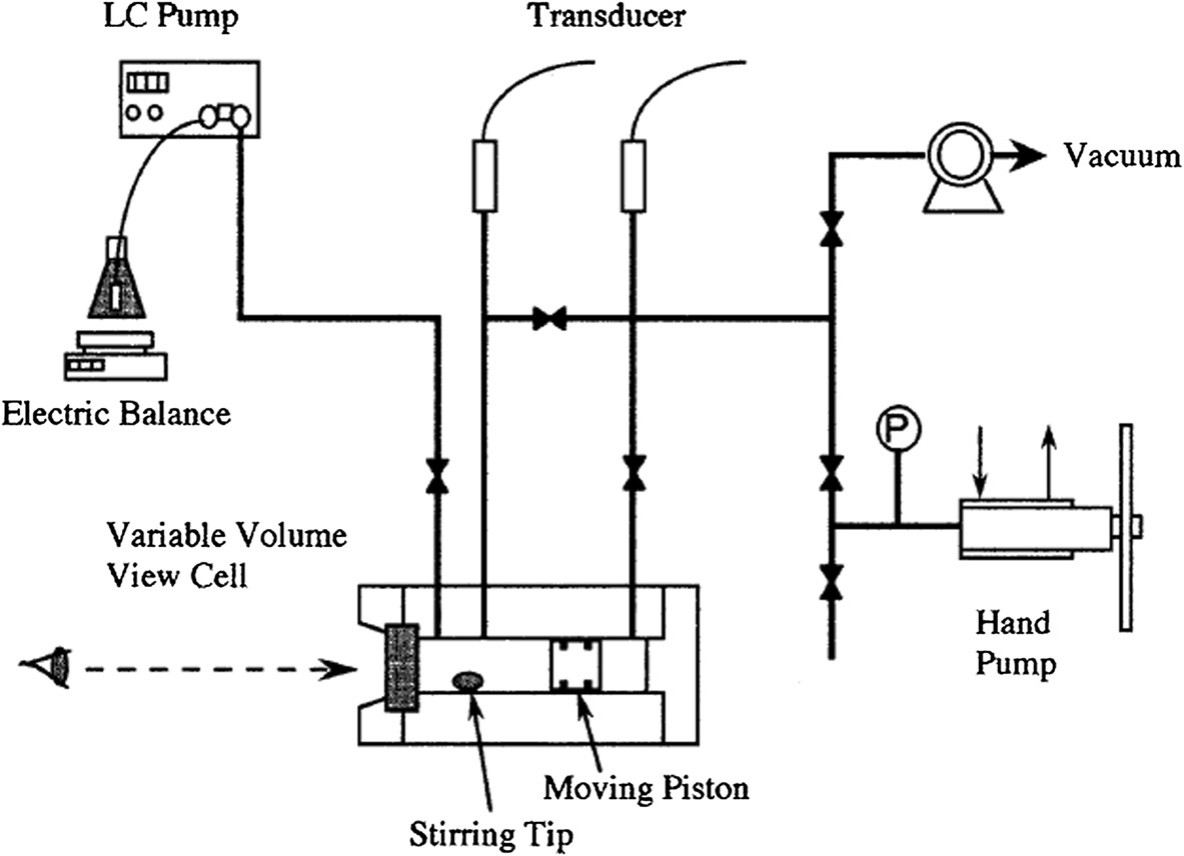
Schematic representation of the scRPE apparatus.

Developed by Otake et al. [41], the supercritical reverse-phase evaporation (scRPE) is a batch process that consists in a constant mix of phospholipids, ethanol, and CO_2_ at a constant temperature (333 K) and pressure (20.0 MPa) values. The temperature value has to be higher than the lipid phase transition in order to ensure the complete dissolution of the lipid in the supercritical phase. Basically, CO_2_ was inserted into a cell with variable volume (depicted in Figure 7) after it was already sealed with ethanol and different amounts of dipalmitoylphosphatidylcholine (DPPC). Then the working temperature and pressure were set and the system was kept in equilibrium for several minutes. After that, an aqueous glucose solution (0.2 mol L^-1^) was added by an HPLC pump with a flow rate of 0.05 mL min^-1^. After the solution was completely added, the system was slowly depressurized forming liposomes with sizes from 0.1 to 1.2 μm with an encapsulation efficiency of 25% for glucose. In addition, the encapsulation efficiency of lipophilic substances was also studied and cholesterol was the model molecule utilized. For this substance, the reached encapsulation efficiency was 63%.

In order to investigate if this method was also compatible with phospholipids other than DPPC, Imura et al. [42] prepared different soybean lecithin-based liposomes. The lipid vesicles produced in this study were constituted of phosphatidylcholine and three different natural lecithins, which are mixtures of PC, phosphatidylethanolamine (PE), phosphatidylinositol (PI), and phosphatidic acid (PA) in different concentrations. Pressure and temperature values were the same as those used by Otake et al. [41]. It was shown that liposomes from different lecithins can be formed, and as expected, their size and shape were dependent on the solubility of the lipid in the supercritical phase. Liposomes constituted by PC presented size diameter varying from 0.2 to 1.2 μm and spherical shape, while vesicles formed by a natural lecithin (32% PC, 31% PE, 17% PI, and 9% PA) were ellipsoidal vesicles with diameter of 0.1 to 0.25 μm.

Based on these previous results, Imura et al. [14] decided to improve the encapsulation efficiency of glucose and the stability of the vesicles adding a different phospholipid to the composition, the dioleoylphosphatidylcholine (DOPC). So, liposomes formed by DOPC or DPPC were prepared with pressures between 130 and 30.0 MPa and temperature of 333 K. It was shown that the maximum glucose-entrapping efficiency for liposomes made of DOPC was 40% (20.0 MPa and 333 K) and 20% for DPPC at the same conditions. It can be noticed that the enhancement of entrapping efficiency was not too significative if this study is compared with other studies of the group [41].

Otake et al. [43,44] simplified the scRPE method in order to enhance the liposome entrapment efficiency. The lipid vesicles were still produced inside a view cell with variable volume; however, the organic solvent was excluded of the mixture, generating an inhomogeneous mixture of phospholipids and aqueous solution at the same parameters utilized for the scRPE method. The system was submitted to magnetic stirring and then pressurized. After the equilibrium period of 40 min, approximately, the system was depressurized and liposomes with mean diameter of 1.5 μm were formed.

### Solid lipid nanoparticles

Created in the 1990s, solid lipid nanoparticles (SLNs) are colloidal particles composed of lipids which are solids in ambient temperature. The term lipid includes triglycerides, partial glycerides, fatty acids, steroids, and waxes. The drug incorporated into SLN is released on a prolonged profile; thus, after administration, a constant concentration of the drug molecule can be maintained in the blood stream. The maintenance of constant plasma levels implies possible reduction of side effects and reduces the frequency of doses of pharmaceuticals. The literature has demonstrated that beyond the composition of lipid matrix, the method of preparation seems to have an important role in the definition of the release mechanism of drug molecule [53-56].

Currently a wide range of techniques for the production of SLN is available. Solvent emulsification/evaporation, high-pressure homogenization, and hot and cold homogenization have been the most cited. The choice of these processes is favored by their feasibility for scaling up to industry production and relatively low overall costs of operation. On the other hand, these traditional methods are multi-step and generally involve high temperature and shear rates, and several cycles at high pressure. These extreme process conditions lead to an increase and heterogeneity of particle size and degradation of the drug. Further, the high kinetic energy content of the obtained particles promotes their coalescence and the presence of organic solvent residues compromises their safety for human use [57].

### Solid lipid particle production by scCO_2_ processing

Considering the broad context on manufacturing limitations of SLN, the supercritical fluid technology appears as a great opportunity to overcome them. Indeed, in this innovative field, the obtention of solid lipid particles at nanometer scale has been a challenging task. Even so, the versatility of supercritical fluid-based plants often offers different solutions for this issue. Table 2 summarizes the different methods applied in the production of solid lipid particles with diversified composition.

**Table 2:** Available works on the production of solid lipid particles by supercritical fluid technology

**Method**	**Lipid composition**	**Active ingredient**	**Particle size**	**Ref**
Supercritical fluid-based coating	Gelucire^®^ 50/02	Bovine serum albumin	125 to 500 μm	[58]
	Trimyristin	Bovine serum albumin	~50 μm	[58]
Supercritical fluid extraction of emulsions	Gelucire^®^ 50/13, tripalmitin, or tristearin	Indomethacin or ketoprofen	~30 nm	[59]
Supercritical co-injection process	Precirol^®^ ATO 5	Pseudoephedrine chlorhydrate or bovine serum albumin	~60 μm	[60]
Particles from gas-saturated solutions	Hydrogenated palm oil	Theophylline	~3 μm	[61]
	Glycerylmonostearate	Caffeine	~5 μm	[62]
	Glycerylmonostearate and Cutina^®^ HR	Caffeine, glutathione, or ketoprofen	NM	[63]
	Precirol^®^ ATO 5 and/or Gelucire^®^ 50/13	Trans-chalcone	1 to 6 μm	[64]
	Glycerylmonostearate and Cutina^®^ HR	Ketoprofen	NM	[65]
	Precirol^®^ ATO 5	Ascorbic acid	~2 μm	
	Myristic acid or tripalmitin	Ibuprofen	2 to 4 μm	[66]
	Beeswax	Menthol	~2 to 50 μm	[67]
	Ceramide 3A, cholesterol, and Radiacid^®^	-	200 to 500 nm	[68]
	Tristearin and Epikuron 200^®^	Insulin or recombinant human growth hormone	~197 nm	[69]
	Tristearin and Epikuron 200^®^ or tristearin, Epikuron 200, and PEG	Insulin	80 to 120 nm	[70]
	Tristearin, Epikuron 200^®^, and oleic acid	Magnetite nanoparticles	200 to 800 nm	[71]
	Tristearin and Epikuron 200^®^	Ribonuclease A functionalized or not with PEG_5000_	4 to 13 μm	[72]

NM, not measured.

#### ***Supercritical fluid-based coating technique***

**Figure 8:**
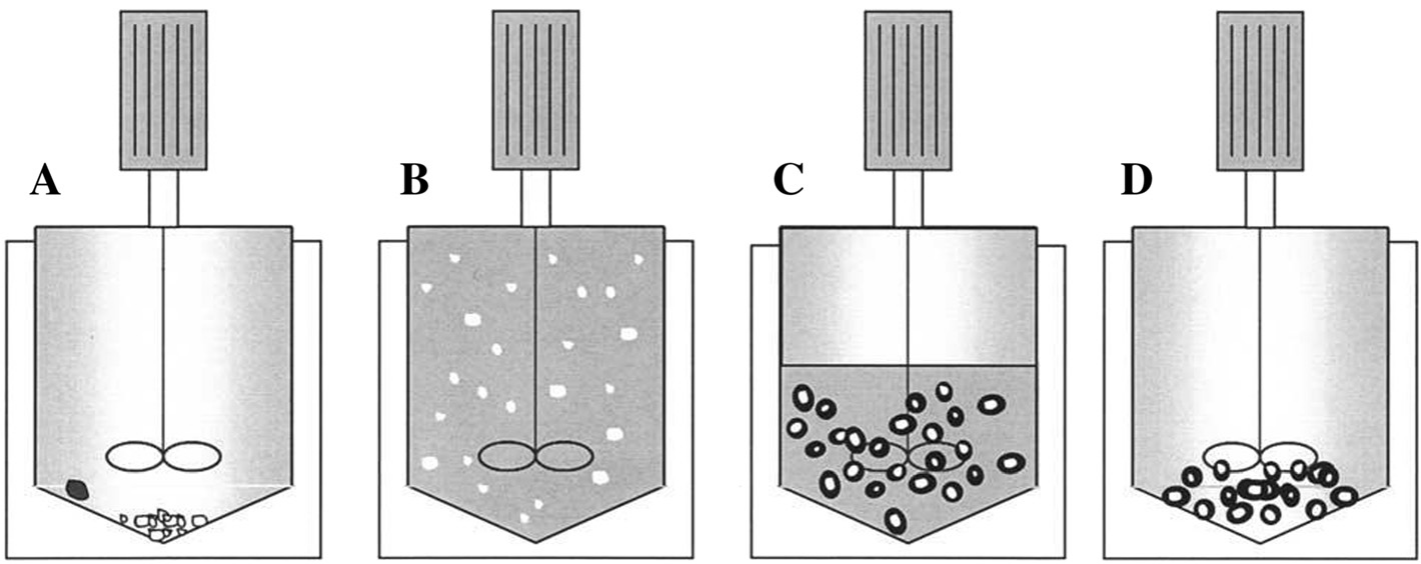
**Schematic representation of the coating process developed by Ribeiro dos Santos et al.**[58]. **(A)** Filling step: BSA crystals (white) and lipid material (black). **(B)** Solubilization of lipid in scCO_2_ with dispersion of insoluble BSA crystals. **(C)** Decompression phase with lipid deposition on BSA. **(D)** Coated particles are obtained.

Benoit et al. [73] developed a relatively rapid, simple, and totally solvent-free technique for coating drug particles with solid lipid compounds. The same group demonstrated the performance of its proposed method by encapsulation of bovine serum albumin (BSA) crystals with trimyristin and Gelucire^®^ 50/02, a commercial mixture of glycerides and fatty acid esters [58]. The scheme of the apparatus used is depicted in Figure 8. The mechanism of coated particle formation is composed of the total solubilization of the solid lipid into scCO_2_ in a thermostatized high-pressure mixing chamber loaded with BSA crystals. After 1 h of mixing, the chamber was depressurized with passage of scCO_2_ to gas state with consequent precipitation of the lipid on the crystal surfaces. This work was described with more details in three other articles [74-76]. As Gelucire is a mixture, it does not crystallize, allowing a uniform coating of BSA, while trimyristin crystallizes and forms a needle-like structure around BSA crystals leading to a burst release from the particles. However, this method is restricted to lipids with considerable solubility into scCO_2_, and the particle size is dependent on the size of the original BSA crystals. Thus, to obtain solid lipid particles with a narrow range of size distribution, the bulk drug has to be processed by an additional technique increasing the final cost of the whole process.

#### ***Supercritical fluid extraction of emulsions***

The super critical fluid extraction of emulsion (SFEE) technique, developed by Chattopadhyay and co-workers [77], is composed of coupling of a conventional method for oil-in-water (o/w) emulsion obtention and subsequent extraction process by scCO_2_. The emulsion is typically prepared by dissolution of a solid lipid and the drug into an organic solvent. This organic solvent is dispersed into the aqueous phase by a homogenizer, using a certain surfactant for stabilization. Then, the emulsion is bombed until atomization through a nozzle and submitted to an extraction of the organic solvent by scCO_2_ in countercurrent flux with consequent solidification of lipid droplets and collection of aqueous suspension of solid lipid particles [77,78].

Compared to traditional methods, this technique brings the advantage of improving the removal of the internal organic phase without affecting the emulsion stability, with shorter processing time and innocuous residual solvent concentration in the final product. Furthermore, due to diffusivity features of scCO_2_, the mass transfer on solvent removal is more efficient in comparison to conventional methods, which lead to a more consistent particle size distribution, avoiding aggregation. Taking into account the smaller droplet size in the primary emulsion, smaller SLNs are obtained; the production of the emulsion represents a pivotal step for achievement of SLN with narrow size range [79-81].

**Figure 9:**
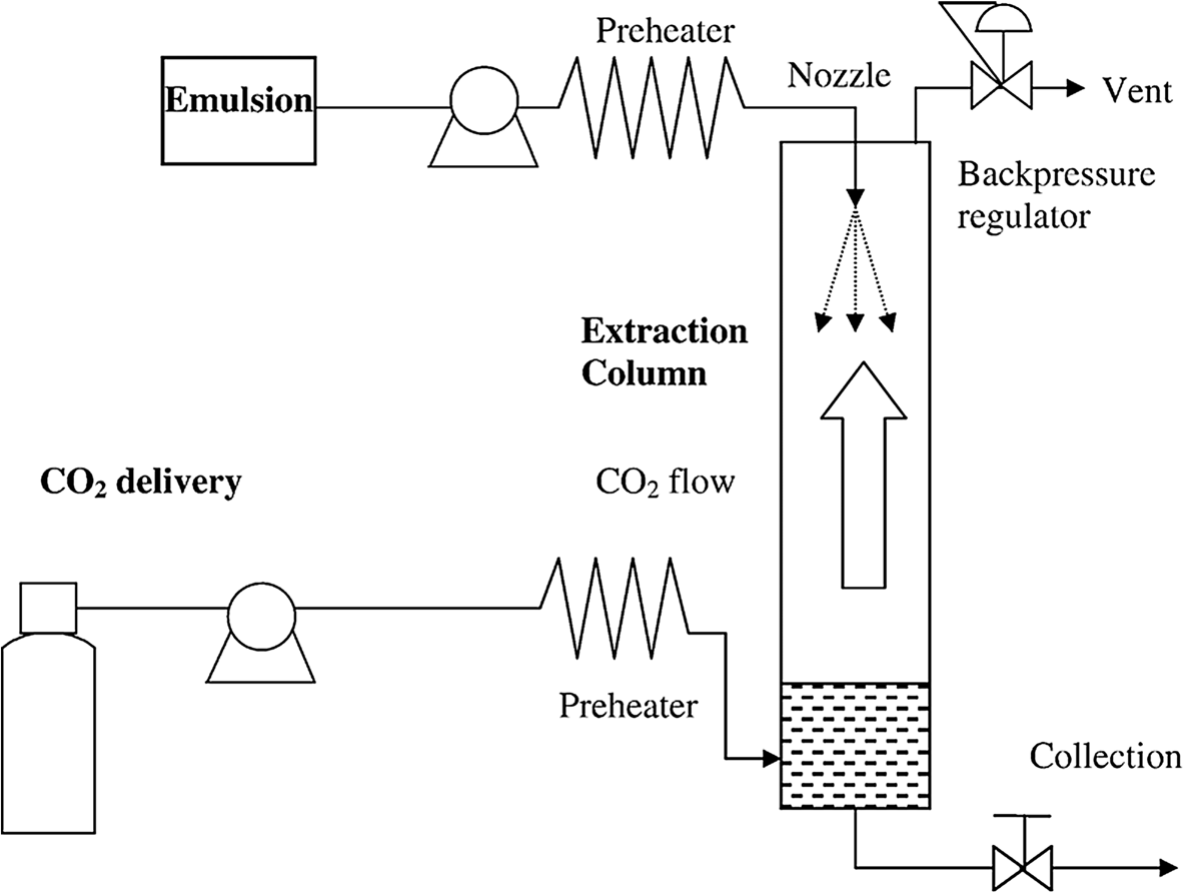
**Extraction system used in the SFEE process developed by Chattopadhyay et al.**[59].

Figure 9 describes the extraction plant used by Chattopadhyay et al. [59] for production of SLN constituted of tripalmitin, tristearin, or Gelucire 50/13. After preparation of an o/w emulsion with oil phase composed of the drug and lipid dissolved in chloroform, the solvent was extracted with scCO_2_ countercurrently at a flow rate of 40 g min^-1^. SLNs with a mean diameter of 30 nm were obtained, however, with a bimodal population composed of a primary peak ranging from 20 to 60 nm and a secondary peak (<10%) of about 200 nm. A residual chloroform concentration of <20 ppm was detected that is in accordance with the International Conference on Harmonization guidelines whose limit for this solvent is 60 ppm [82].

Earlier, by using the SFEE plant already cited above (Figure 8), Shekunov et al. [81] performed micronization studies on cholesterol acetate and griseofulvin and evaluated possible important factors for definition of particle size that can be taken in consideration for SLN production. It was observed that the droplet size, drug concentration, and solvent content are the major factors with significant influence on particle size. Naturally, when the size of o/w emulsion droplets is smaller, smaller particles can be obtained. Thus, the stabilization of the emulsion by a surfactant is highly important owing to its capability to guarantee the maintenance of small droplets and avoidance of aggregation events [83]. On the other hand, the partial interaction of the drug molecule with the aqueous media may promote the interaction among droplets that aggregate and form larger particles. In addition, considering that supersaturation in emulsion droplets is important for the formation of small particles, the increase of solvent content promotes increase in growth rate. These conclusions also correlate with studies conducted with PLGA nanoparticles [84].

#### ***Supercritical co-injection process***

**Figure 10:**
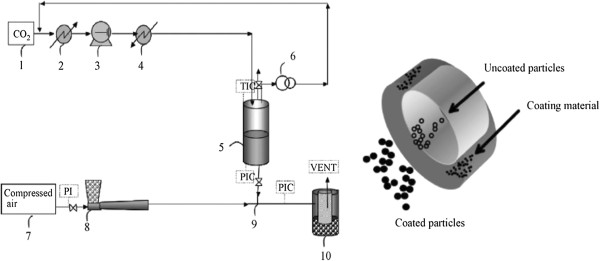
**The supercritical co-injection process.** (Left) Schematic representation of the supercritical co-injection process: (1) CO_2_cylinder, (2) cooler, (3) pump, (4) heater, (5) saturation vessel, (6) high-pressure vessel, (7) valve, (8) pneumatic conveying, (9) co-injection advice, (10) gas/solid separation filter, (PI) pressure indicator, (PIC) pressure indicator and controller, (TIC) temperature indicator and controller, and (VENT) Venturi. (Right) The co-injection device [60].

Developed by Calderone and colleagues [85], the co-injection process was presented as a new way for the obtention of solid lipid microparticles. As described in Figure 10, firstly, a solid lipid is melted under its normal melting point due to the plasticizing effect exercised by solubilization of a pressurized gas. Second, the expansion of the gas-saturated melted lipid phase causes its pulverization. This pulverization occurs in a custom-designed co-injection device, where particles of uncoated drug are conveyed by a Venturi system at the same time. The co-injection provides the coating of the drug particles [60].

This method presents the advantage of maintaining the active component in a different reservoir than that used for the coating material; thus, the drug component may be exposed to ambient temperature conditions which prevent its degradation. By using Precirol^®^ ATO5 for coating of pseudoephedrine chlorhydrate (PSE) and BSA, the method was tested by Calderone et al. [60]. The effective coating of the particles, with significant retarding of drug release in aqueous media, was demonstrated. Meanwhile, the observed drug release cannot be classified as prolonged because of the relatively short time for release of 100% of the entrapped PSE (50 min) and BSA (30 min). In pre-tests carried out with glass beads for validation of this method, it was found that aggregation events of beads smaller than 20 μm were very common. It brings an important limitation on achieving particles in nanometer scale.

#### ***Particles from gas-saturated solutions***

**Figure 11:**
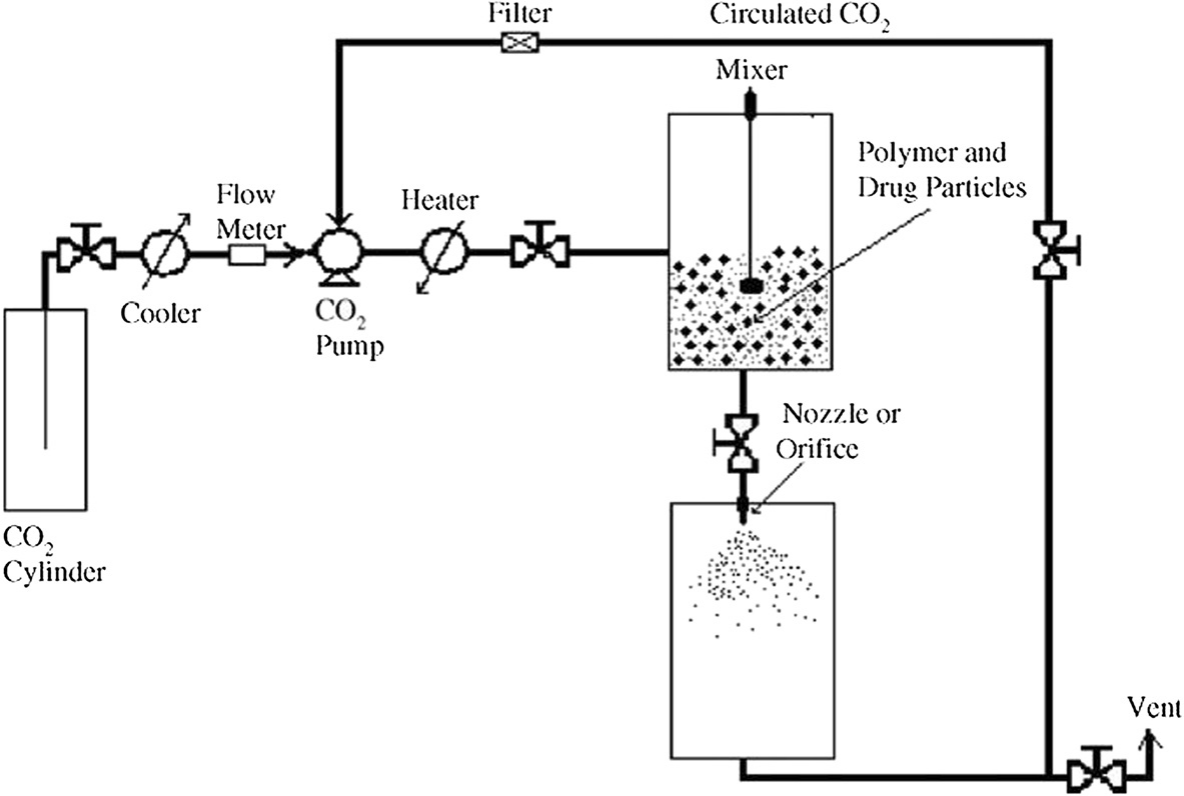
**Example of PGSS plant for particle formation for drug-loaded particles **[46].

Among the available techniques for SLN production by supercritical fluid processing, particles from gas-saturated solutions (PGSS) have been shown as the most interesting. Also known as supercritical melt micronization process [86], PGSS is a completely solvent-free process where a solid is melted in a highly pressurized vessel pressurized by a compressed gas. Figure 11 demonstrates a generic scheme of a PGSS plant used for drug-loaded polymeric and lipid particles. There, gas-saturated solution is expanded through a nozzle, and due to the Joule-Thompson effect, it is rapidly cooled down leading to formation of SLN [87,88]. In addition to all advantages of supercritical fluid technology, PGSS can produce directly powdered formulations, requires the use of small-volume pressurized equipment, demands relatively low amounts of CO_2_, easily performs the recovery of the product and the gas, and is useful for the production of polymer powder or the entrapping of active ingredients in polymer matrices. This process already runs in plants with the capacity of some hundred kilograms per hour [89,90]. Another great advantage of the PGSS technique resides in the plasticizing effect of scCO_2_ when diffused into a polymer or lipid matrix which allows their melting under mild temperatures, becoming feasible for drug processing [91]. Further, PGSS usually provides particles (μm or nm) with uniform narrow size range of particular interest [4].

However, the mechanisms of particle formation are not completely understood. Several studies have been conducted for modeling of particle formation in PGSS, and it was found that the expansion process is composed of atomization and nucleation/crystallization phenomena [92,93]. Briefly, atomization can be defined as the disruption of a liquid jet in fine particles during expansion [94]. Further, nucleation describes the formation of CO_2_ bubbles inside the fresh droplets of a mixture of molten lipid and drug due to transition to gaseous state of the supercritical fluid in the expansion unit, and crystallization involves the solidification of the particle surface and subsequent inner lipid matrix under decrease of temperature due to the Joule-Thompson effect [95].

Studies have demonstrated that nozzle diameter, pre-expansion pressure and temperature, and flow rate of carbon dioxide represent four of the most important factors for defining the size, shape, and physical state of the particles [96]. It has been found that when the saturation pressure is larger, a larger carbon dioxide diffusion into polymer or lipid matrix is achieved, while there is an inverse relationship between scCO_2_solubilization and saturation temperature [97]. The high content of scCO_2_ favored by high saturation pressure makes the nucleation process occur faster than crystallization of surface during the expansion step, leading to formation of small particles. However, the higher the scCO_2_ content is, the more violent is the disruption of the lipid matrix with potential formation of shapeless particles. This is not a desirable effect considering that irregularly shaped particles commonly present a burst release of the active compound [95].

**Figure 12:**
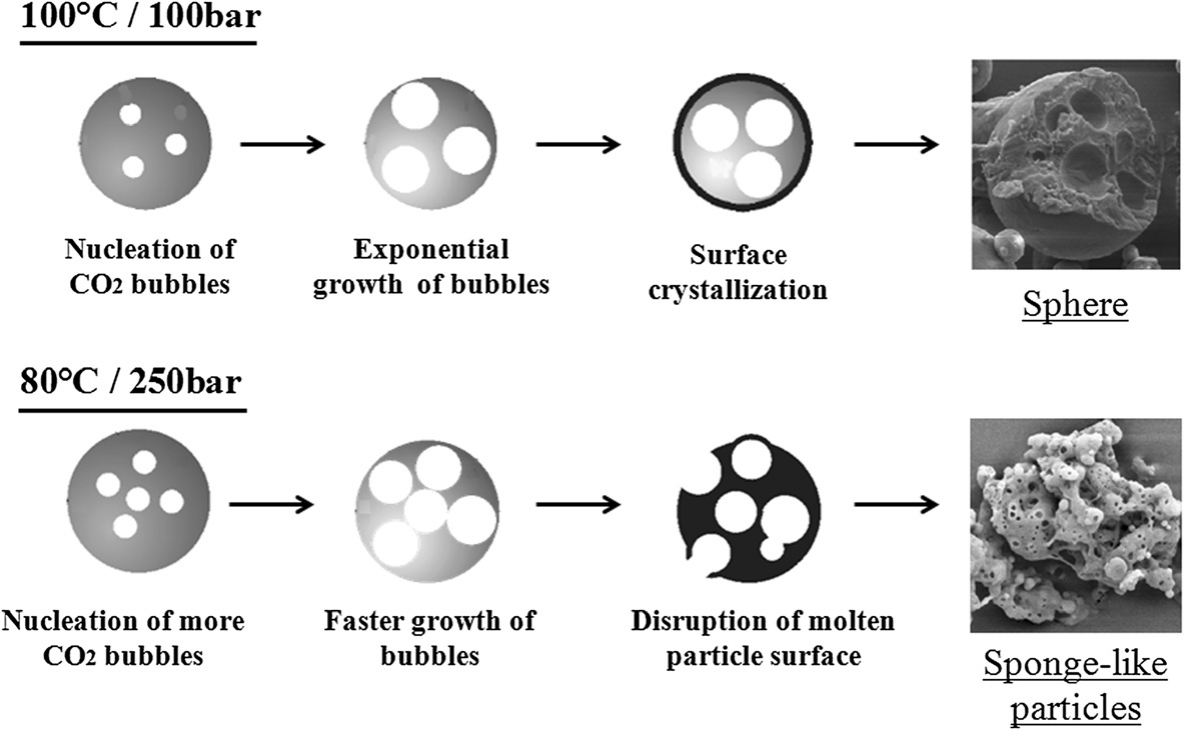
**Different results obtained under different operation conditions in a PGSS method for production of PEG-600 particles.** Adapted from Kappler et al. [95].

In the case of temperature, the opposite effect on particle size is observed, i.e., the particle size increases with increasing temperature above the melting point of the carrier material. This can be explained by the decrease of scCO_2_ solubility upon increasing temperature. Thus, with lower fluid content in the particles, the crystallization of the particle surface occurs faster than CO_2_ bubble formation which leads to retention of the gas and less disruption events resulting in obtention of larger particles. This phenomenon is readily observed when the selected saturation temperature is already below the lipid or polymer melting point [98]. Figure 12 presents a scheme with different particles obtained with different operation conditions in a work performed by Kappler and colleagues [95].

Seeing the wide range of available lipids and drug molecules, the operation conditions are unique depending upon the specific system. Rodrigues et al. [61] produced microcomposite lipid particles composed of hydrogenated palm oil entrapping theophylline by PGSS. Solid lipid particles of about 3 μm were obtained by selecting the conditions of 333 K and a range of 12 to 18 MPa for the mixing step and a nozzle diameter of 25 μm. His group observed that increase of pre-expansion pressure leads to formation of more spherical and larger particles. On the other hand, burst release of theophylline from the particles was detected.

In a similar PGSS plant and with the same pre-expansion operation conditions, Wang et al. [99] achieved trimyristin and tripalmitin particles of about 2 μm loaded with ibuprofen. However, a 100-μm-diameter nozzle was used, indicating that the type of lipid and saturation time also have a significant role in particle size definition. Equipped with an 80-μm-diameter nozzle and under the same pre-expansion conditions, the same authors showed less attractive results from lipid particles synthesized with beeswax and menthol. A multimodal population of particles ranging from 45 to 180 μm was obtained [100]. By application of similar conditions, Sampaio de Sousa and colleagues [62] achieved glyceryl monostearate microparticles of about 5 μm loaded with caffeine, though, owing to the hydrophilicity of caffeine, it was necessary to use water as co-solvent. Further studies on the formulation under 13 MPa and 345 K with the addition of Cutina^®^ HR and titanium dioxide, an anticaking additive, showed that the low affinity of hydrophilic compounds such as caffeine and glutathione resulted in a low payload and a burst release. Otherwise, a lipophilic compound, ketoprofen, presented a high entrapment rate and sustained release (*t*_2h_ = 20%) [63].

**Figure 13:**
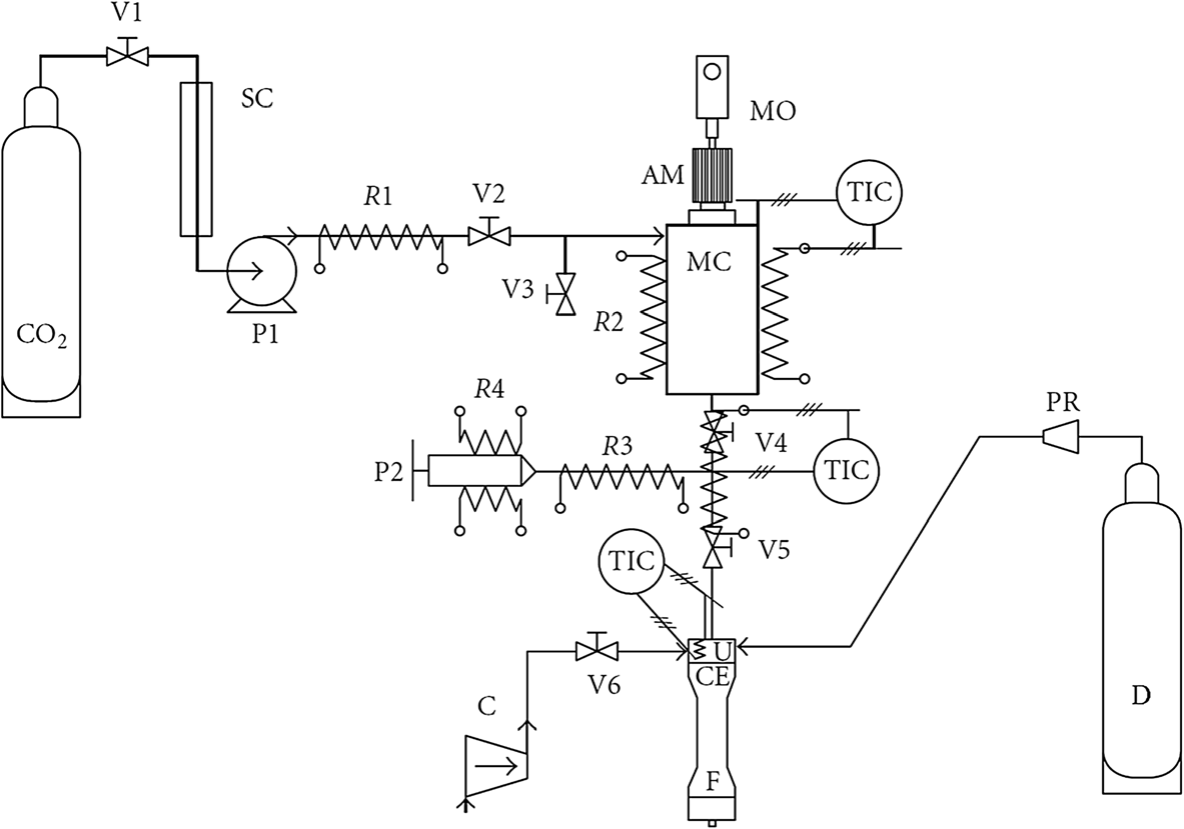
**Schematic of the modified PGSS apparatus adapted from Vezzù et al**. [71]. MO, electric motor; AM, stirrer; MC, mixing chamber; U, nozzle; CE, expansion chamber; F, filter; *R*1 to *R*4, electric resistances; SC, heater exchanger; P1, pump; P2, manual syringe pump; V1 to V6, on-off valves; PR, pressure reducer; C, air compressor; D, synthetic air or nitrogen cylinder; TIC, temperature indicator and controller.

Intending the successful achievement of solid lipid particles in nanometer scale, Bertucco et al. [101] developed a modified PGSS method in which the particle formation is assisted by an auxiliary gas, synthetic air, nitrogen, or the combination of both, as depicted in Figure 13. This modification enabled the obtention of submicron-sized lipid particles. Based on this method, at pre-expansion conditions set at 15.0 MPa and 313 K and a 100-μm nozzle, SLNs loaded with insulin or human growth hormone (HGH) were produced with a lipid matrix composed of phosphatidylcholine and tristearin, spherical shape, a mean diameter of 197 nm, and a mean loading efficiency of 57% and 48% for insulin and HGH, respectively [69]. Taking into account the hydrophilic nature of some bioactive compounds like insulin, HGH and other proteins, dimethyl sulfoxide (DMSO) is commonly used to facilitate their homogeneous dispersion in the lipid mixture [69,70,72]. The addition of DMSO in the formulation promoted an increase in loading efficiency to 80%, with values of residual solvent below 20 ppm [70]. By using of the same saturation conditions, SLNs based on tristearin and magnetite nanoparticles (Fe_3_O_4_) of about 200 nm were also produced, and the loading capacity was slightly increased with the addition of phosphatidylcholine [71].

The good results obtained by Bertucco and colleagues in entrapping hydrophilic compounds in SLN, with maintaining of a sustained release, reveal the necessity of selecting the correct emulsifier and/or co-solvent. Without them, not only a low encapsulation rate is achieved, but during particle formation in the expansion unit, a phase separation between the drug and the lipid may occur. This condition favors the deposition of the drug on the particle surface generating a burst release [63].

## Conclusions

A large number of supercritical fluid processes for the production of different drug delivery systems were found in the literature, which can demonstrate that this technology is suitable for the design of lipid micro- and nanoparticles, namely liposomes and solid lipid nanoparticles. Furthermore, it can be seen that the use of supercritical fluid-based processes enables more homogenized particles and reduces the environmental impact. Despite the promising features of these techniques, the scalability outside scientific laboratories and industrial implementation of these processes are still expensive, limiting the industrial production of these particles using these fluids.

## Competing interests

The authors declare that they have no competing interests.

## Authors’ contributions

IES surveyed the data related to liposome production by supercritical fluid technology, whereas ASP surveyed the data related to solid lipid particles. RF supervised and corrected the structural information of the cited plants, and ECA conceived of the study and participated in its design and coordination as well as reviewed the experimental characterization notes. All authors read and approved the final manuscript.
